# Diagnostic performance of routine blood parameters in periodic fever, aphthous stomatitis, pharyngitis, and adenitis syndrome

**DOI:** 10.1002/jcla.24934

**Published:** 2023-07-10

**Authors:** Hakan Onur, Arzu Rahmanali Onur

**Affiliations:** ^1^ Department of Pediatrics Memorial Private Diyarbakir Hospital Diyarbakir Turkey; ^2^ Department of Medical Microbiology Gazi Yasargil Education and Research Hospital Diyarbakir Turkey

**Keywords:** C‐reactive protein, laboratory parameters, mean platelet volume, neutrophil‐lymphocyte ratio, PFAPA syndrome

## Abstract

**Background:**

We aimed to investigate the difference between PFAPA and streptococcal tonsillitis (Strep Pharyngitis) by using blood parameters. We want to evaluate the relationship between periodic fever, aphthous stomatitis, pharyngitis, adenitis (PFAPA) syndrome, and tonsillitis by using NLR.

**Methods:**

The data of 141 pediatric patients who had applied to our clinic between October 2016 and March 2019 and were diagnosed with PFAPA syndrome and tonsillitis were reviewed from hospital records. The demographic data of the study group were recorded, as were their WBC, neutrophil, and lymphocyte counts, NLR, and MPV values, which are obtained by proportioning these two counts.

**Results:**

CRP and ESR values were significantly higher in the PFAPA group (*p* = 0.026 and *p* < 0.001, respectively). No significant difference was determined between the groups in terms of platelet count or lymphocyte count. Receiver operating curve analyses were calculated. The AUC was 0.713 ± 0.04 according to age, and the CRP was 0.607 ± 0.04 (95% confidence interval). Using a cutoff point of >49 months for age, the sensitivity was 0.71 and the specificity was 0.67.

**Conclusion:**

With simple laboratory parameters, PFAPA syndrome can be differentiated from a diagnosis of tonsillitis. This may reduce the costs associated with unnecessary antibiotic use. However, these findings still need to be confirmed by other future studies.

## Graphical abstract

Use of CRP and ESR in the model used to differentiate PFAPA and tonsillitis groups with blood markers.

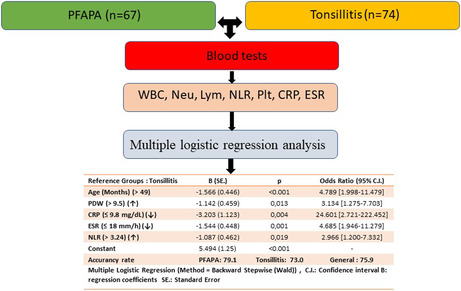

## 1 INTRODUCTION

The most frequent form of fever in children is periodic fever, aphthous stomatitis, pharyngitis, and adenitis (PFAPA) syndrome, with the majority of cases seen before the age 5.^1^ These four primary symptoms serve as the basis for the disease's name. Only when all other auto‐inflammatory disorders and infections are ruled out can PFAPA, one of the most prevalent auto‐inflammatory diseases in children, be diagnosed.^2^ It was first defined in 1987 by Marshall et al.^3^, ^4^ The diagnostic criteria that were developed have very little specificity. Diagnostic challenges lead to delayed or incorrect diagnoses. Late diagnosis can have a negative impact on the patient's quality of life, whereas misdiagnosis results in the overuse of antibiotics. The diagnosis of PFAPA may be challenging in persons who have underlying disorders that predispose them to upper respiratory tract illnesses such as allergic rhinitis and recurrent fever. For PFAPA syndrome, there is no test for diagnosis. It is possible to make a diagnosis with close monitoring and clinical suspicion.^5^ Auxiliary diagnostic tools must be created due to the challenges in diagnosis.

Major acute phase protein, C‐reactive protein (CRP), is a pentraxin family member and an essential component of the immune system.^6^ CRP is still the biomarker most frequently utilized and dealt with, despite the fact that novel biomarkers are being used in several research studies. There are few noninvasive, broadly applicable biomarkers with high diagnostic reliability during the childhood. Studies linked to noninvasive techniques have been carried out in the most recent research, giving birth to fresh expectations. In order to conduct a diagnose, complete blood count (CBC) and mean platelet volume (MPV) data are currently employed. The metrics that can be easily acquired as part of a CBC are the neutrophil‐lymphocyte ratio (NLR) and MPV. NLR is calculated by dividing the absolute neutrophil count absolute lymphocyte count. It has been proposed as a sign of systemic inflammation.^7^ It has been used in the differential diagnosis of inflammatory diseases from the neonatal period.^8^ In disorders including familial Mediterranean fever, systemic lupus erythematosus, and juvenile idiopathic arthritis that are linked to systemic auto‐inflammation, NLR has been investigated, and it has been proposed that this ratio can indicate the activity of the disease and the inflammatory response.^9^

In order to diagnose and track inflammation, NLR and MPV data are now employed. NLR has been utilized in adult research to assess the presence and severity of sepsis.^8^, ^9^ Inflammation is indicated by this ratio. We aimed to investigate the difference between PFAPA and Strep Pharyngitis by using blood parameters. We want to evaluate the relationship between PFAPA and tonsillitis by using NLR. As far as we know, PFAPA is an additional autoinflammatory condition for which NLR or MPV have not been studied to date.

## 2 MATERIALS AND METHODS

The pediatric outpatient clinic of the Diyarbakır Memorial Hospital Pediatrics Clinic Hospital served as the setting for this prospective observational study, which was carried out between October 2016 and March 2019. We enrolled 67 consecutive PFAPA patients, who were being treated at our hospital or other related facilities. We chose individuals who had long‐term recurring fever and elevated CRP levels during flare‐ups. Patients with infections, such as Epstein–Barr virus (EBV) infection, systemic chronic disorders, such as hematological, cardiac, pulmonary, hepatic, and renal diseases, those with tumors, immunological weakness, or sepsis, were excluded from the study.

By using the diagnostic standards of Marshall et al.^4^ and Thomas et al.,^10^ PFAPA was diagnosed in these individuals. The inclusion criteria were (1) a physical examination diagnosis of exudative tonsillitis with associated fever; (2) a Marshall criteria diagnosis of PFAPA with at least four episodes; (3) a positive throat culture diagnosis of Strep Pharyngitis; and (4) a physical examination diagnosis of exudative tonsillitis without associated fever. Peritonsillar abscess, known immunodeficiency or chronic illnesses, lack of a precise diagnosis, and missing data for MPV, ESR, CRP, or CBC parameters in medical records were the exclusion criteria. In patients with challenging diagnostics, we resorted to serum cytokine profiles and neutrophil activity during attacks. Patients with unclear diagnosis were excluded from the study. This research was authorized by the local ethics committee and carried out in accordance with the Declaration of Helsinki (Approval date: 05.03.2021 and number: 706). We evaluated 74 individuals with Strep Pharyngitis with fever, in order to compare PFAPA with infectious diseases that have comparable clinical courses and symptoms (over 1 time per year). An expert doctor (H.O.) made the final diagnosis for the PFAPA patients after carefully examining all the available data and doing a physical examination. In order to avoid bias, all diagnoses were made by a single physician.

The hospital's medical database provided patient laboratory and clinical data. All patients had a “rapid streptococcal antigen assay” as well as studies on their CBC, CRP, and ESR.

### 2.1 Blood sampling and assays

For CBC and flow cytometry analysis, peripheral blood was collected in a tube containing ethylene diamine tetraacetic acid (EDTA) (1.2 mg/mL) (Sysmex Xt 2000, Japan). The ratio of neutrophil count to lymphocyte count was used to compute the NLR. The patients' White blood cell count (WBC), absolute neutrophil and lymphocyte counts, MPV, CRP, and ESR levels, as well as how many episodes they had had before admission, were noted. MPV, a measure of the platelets' average size and level of maturation, was also noted.

An automated dynamic analyzer (True line 200, Turkey) was used to measure ESR, while a gold standard digital quantitative analyzer (Cobas Integra 400) was used to measure CRP. Tubes containing liquid EDTA were used to measure CBC and ESR, while tubes containing a gel separator were used to measure CRP.

### 2.2 Diagnosis of Strep Pharyngitis

It is advised to employ a diagnostic scoring system based on Centor or McIsaac to determine the likelihood that tonsillitis was brought on by hemolytic streptococci. A positive score of 3 should trigger a pharyngeal swab, fast test, or culture to detect hemolytic streptococci if therapy is being considered.^11^ It is not necessary to perform routine blood testing for acute tonsillitis. After acute Strep Pharyngitis, standard cardiovascular diagnostics such as pharyngeal swabs or other routine blood tests, urinalyses, or EKG do not need to be repeated. The presence of positive growth in a throat culture, allows for the identification of group A beta hemolytic streptococcus.^11^

### 2.3 Statistical analysis

The applications MedCalc 14 (Acacialaan 22) and SPSS 25.0 (IBM Corporation) were used to analyze the variables. The Shapiro–Wilk Francia test and the Levene test were used to assess the homogeneity of variance and the conformance of univariate data to the normal distribution. The Independent‐Samples *T* test and the Bootstrap findings were used in the comparison of two independent groups based on quantitative variables, the Mann–Whitney *U* test and the Monte Carlo results were used. Using the Monte Carlo Simulation approach, the Pearson Chi‐Square test was used to compare categorical variables to one another. Sensitivity, specificity, positive and negative predictive values, as well as the connection between the cutoff value and the actual classification, were examined and expressed via ROC (receiver operating curve) curve analysis. To ascertain the cause‐and‐effect connection between the groups and the explanatory variables, the ackward approach was used with the logistic regression test. The tables presented quantitative data as mean (standard deviation) and median (minimum/maximum), whereas categorical variables were represented as *n* (%). A *p*‐value of <0.05 was regarded as significant during the 95% confidence level analysis of the variables.

#### 2.3.1 Power analysis

In our study, power analysis was performed for patients who were taken at regular intervals. Since the study reached 80% power level in 141 patients, the study was conducted on 141 patients.

## 3 RESULTS

A total of 141 children were involved in the study: 67 PFAPA patients and 74 with Strep Pharyngitis. Patients with PFAPA had smaller ages than tonsillitis group (*p* < 0.001), (Table 1). The median age was 43 months (17–93) in the PFAPA group and 61 months (18–154) in the tonsillitis group. There was no significant difference between the groups in terms of the distribution of gender (*p* > 0.05). All PFAPA patients had a negative rapid streptococcal antigen assay result. The number of episodes before admission ranged between 2 and 8.

**TABLE 1:** Laboratory evaluation of study groups.

	PFAPA (*n* = 67)	Tonsillitis (*n* = 74)	*p*
	Mean ± SD	Mean ± SD	
White blood cell (10^3^/mm^3^)*	14.040 (4.41)	15.13 (6.19)	0.231^a^
Hemoglobin (Hb) (g/dL)*	12.07 (0.97)	12.44 (0.94)	0.027^a^
Platetet (PLT) (10^3^/mm^3^)*	338.33 (105.19)	347.34 (102.46)	0.595^a^
Red cell distribution width (RDW)*	14.56 (1.04)	14.19 (1.55)	0.096^a^
	**Median (IQR)**	**Median (IQR)**	
Platelet distribution width (PDW)**	9.5 (8/14.9)	10.5 (8.3/14.8)	0.095^b^
Neutrophil count (10^3^/mm^3^)**	8.820 (1.380/23.170)	9.500 (1.820/31.820)	0.233^b^
Lymphocyte count (10^3^/mm^3^)**	2.860 (1.110/5.770)	2.760 (840/7600)	0.170^b^
C‐reactive protein (mg/dL)**	32 (9.26/225)	27.13 (0.21/133.9)	0.026^b^
Erythrocyte sedimentation rate (ESR) (mm/h)**	24 (5/66)	15 (5/77)	<0.001^b^
Mean platelet value (MPV) (fL)* *	8.7 (7.3/11)	9.1 (7.7/11.4)	0.196^b^
NLR**	2.95 (0.31/14.3)	3.55 (0.26/18.08)	0.048^b^

^a^ Independent *t* test (Bootsrap).

^b^ Mann Whitney *U*.

*, *p*, 0.05 and **, *p*, 0.001.

White blood cell count, neutrophil and lymphocyte levels, as well as PLT levels were similar in both groups (Table 1). Laboratory evaluation of study groups was given in Table 1. Although hemoglobin levels were lower in the PFAPA group, CRP levels were higher in PFAPA group (*p* = 0.027 and *p* = 0.026, respectively), which was statistically significant. The NLR rate was greater in the Strep Pharyngitis group, while the erythrocyte sedimentation rate was statistically substantially higher in the PFAPA group (respectively, *p* = 0.001, *p* = 0.048).

**FIGURE 1:**
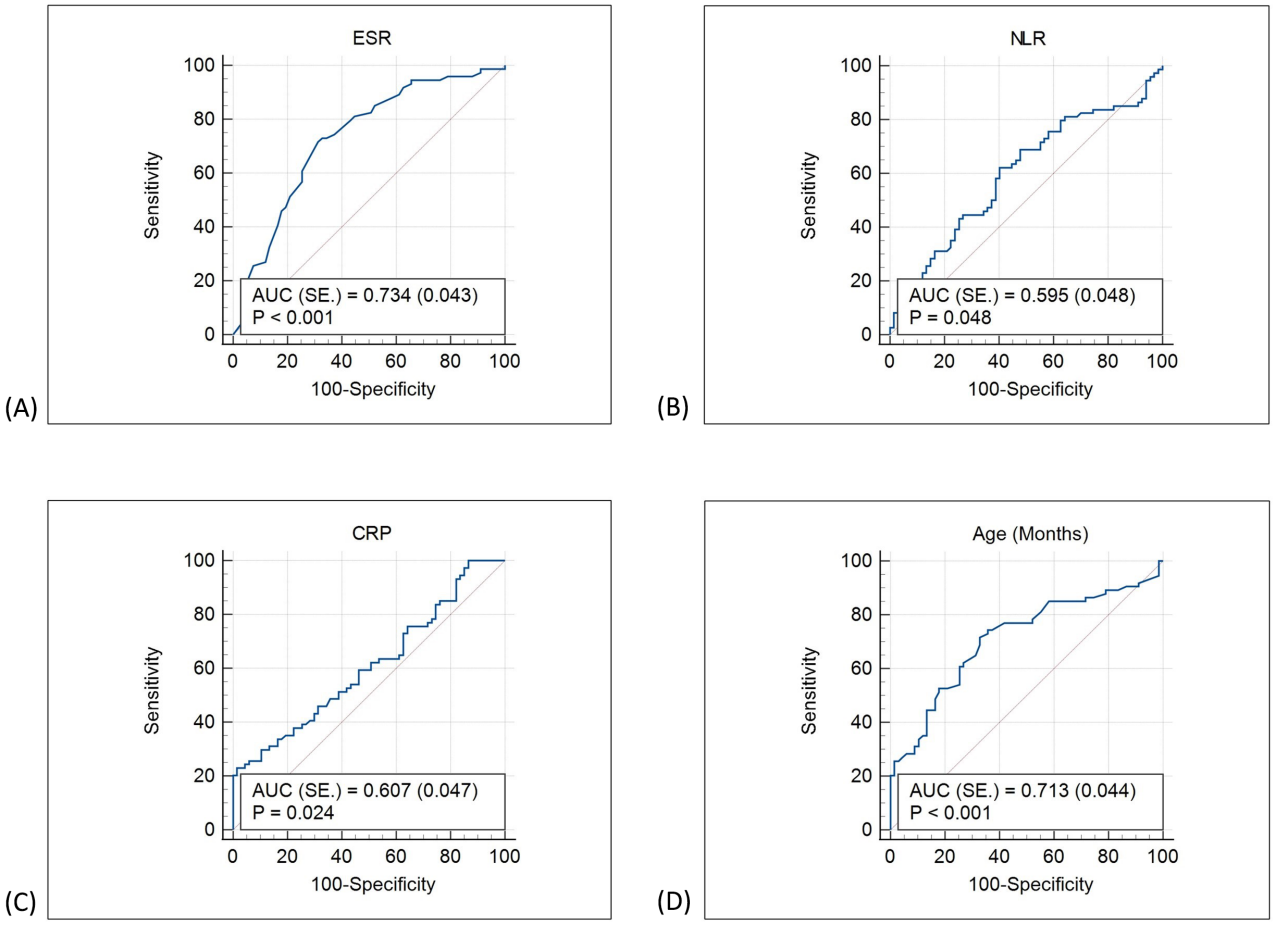
The ROC of mean CRP (A), ESR (B), NLR (C), and age (D). CRP and ESR was better than NLR and can be used for differential diagnosis.

Receiver operating curve analysis for laboratory parameters were given in Table 2. The AUC was 0.713 ± 0.04 according to age and CRP was 0.607 ± 0.04 (95%, confidence interval) (Figure 1). Using a cutoff point of >49 months for age, the sensitivity was 0.71 and the specificity was 0.67.

**TABLE 2:** ROC analysis of laboratory parameters in study groups according to reference group.

Reference streptococal tonsillitis	Cutoff	Sensitivity	Specificity	PPV	NPV	AUC ± SE	*p*
Hb (g/dL)	>11.8	74.3	44.78	59.8	61.2	0.619 ± 0.047	0.012
RDW	≤14.2	66.2	62.69	66.2	62.7	0.652 ± 0.047	0.001
Age (months)	>49	71.62	67.1	70.7	68.2	0.713 ± 0.044	<0.001
PDW	>9.5	68.92	52.24	61.4	60.3	0.581 ± 0.049	0.096
CRP (mg/dL)	≤9.8	22.97	98.51	94.4	53.7	0.607 ± 0.047	0.023
ESR (mm/h)	≤18	71.6	68.66	71.6	68.7	0.734 ± 0.043	<0.001
NLR	>3.24	62.16	59.7	63	58.8	0.595 ± 0.048	*0.048*

*Note*: *p* < 0.001 and *p*: 0.012 statistical significant values are italics.

Abbreviations: AUC, area under the ROC curve; NPV, negative predictive value; PPV, positive predictive value; ROC, receiver operating curve (analysis [Honley & Mc Nell – Youden index J]); SE, standard error.

Our associated variables in the estimation of the groups were found to be age, platelet distribution width (PDW), CRP, ESR, and NLR according to the logistic regression model (*p* < 0.001). According to this model, the accuracy rates of the groups correctly identify the PFAPA group rate as 79.1%, the Strep Pharyngitis group as 73% and the overall accuracy rate as 75.9%. According to this model, our highest correlated variable was CRP, while our least associated variable was NLR. Multilogistic regression analysis results are given in Table 3.

**TABLE 3:** Multiple logistic regression analysis of parameters according to reference group.

Reference groups: streptococal tonsillitis	*B* (SE)	*p*	Odds ratio (95% CI)
Age (Months) (>49)	−1.566 (0.446)	<0.001	4.789 [1.998–11.479]
PDW (>9.5) (↑)	−1.142 (0.459)	0.013	3.134 [1.275–7.703]
CRP (≤9.8 mg/dL) (↓)	−3.203 (1.123)	0.004	24.601 [2.721–222.452]
ESR (≤18 mm/h) (↓)	−1.544 (0.448)	0.001	4.685 [1.946–11.279]
NLR (>3.24) (↑)	−1.087 (0.462)	0.019	2.966 [1.200–7.332]
Constant	5.494 (1.25)	<0.001	–
Accurancy rate	PFAPA: 79.1	Tonsillitis: 73.0	General: 75.9

*Note*: Multiple Logistic Regression (Method = Backward Stepwise [Wald]).

Abbreviations: *B*, regression coefficients; CI, confidence interval; SE, standard error.

The rate of those with CRP ≤9.8 mg/dL in the Strep Pharyngitis group was 24.6 (2.7–222.4) times higher than that in the PFAPA group. The rate of those aged >49 months in the Strep Pharyngitis group was 4.789 times (1.998–11.479) higher than that in the PFAPA group. The rate of cases with an ESR value ≤18 mm/h was found to be 4.685 (1.946–11.279) times higher in the Strep Pharyngitis group than that in the PFAPA group.

## 4 DISCUSSION

A common cause of emergency department visits and one of the conditions that families fear the most is fever. The reason for admission is usually an upper respiratory tract infection. However, symptoms similar to that of upper respiratory tract infection may present, although the underlying causes may be noninfectious and may result in misdiagnosis and unnecessary or incorrect treatment. On the other hand, it has been stated that diseases such as allergic rhinitis, which resembles recurrent upper respiratory tract infection, may also cause difficulties in diagnosis.^12^ PFAPA syndrome as well is one of the important diseases that need to be considered in the differential diagnosis of patients presenting with fever. The disease appears to be more prevalent than assumed. In fact, the annual incidence of the disease in Norway was reported to be 2.3/10,000.^13^ PFAPA syndrome is a clinical diagnosis and recurrent fever in patients should be seen and evaluated by a physician. It is very difficult to distinguish episodes of PFAPA syndrome from infections in children under the age of five.^14^ In this study, we aimed to define laboratory parameters that can be used in the differential diagnosis of Strep Pharyngitis and PFAPA syndrome. 67 patients with PFAPA were compared with 74 tonsillitis cases with similar demographic characteristics. In a study conducted in our country, 607 children with PFAPA syndrome were evaluated.^15^ They found the median age at the time of the most recent attack was 66 (24–168; IQR 48–84) months, and the median illness duration was 40 (4–132; IQR 27.5–60) months.^15^ The majority of the patients experienced fever (100%), which was followed by pharyngitis/exudative tonsillitis (594, 97.9%), aphthous stomatitis (308, 50.7%), cervical lymphadenopathy (278, 45.8%), stomach discomfort (249, 41%), and arthralgia (228, 37.6%).^15^ However, we did not evaluate the clinical findings. Hemoglobin value was higher in the tonsillitis group, while CRP and ESR values were lower in the tonsillitis group. In particular, the sensitivity where age was >49 months in terms of tonsillitis was 0.71, and the sensitivity of ESR was 0.71. There are no specific laboratory findings of PFAPA syndrome. Although there are diagnostic criteria for PFAPA syndrome, they are not sufficient to prevent misdiagnosis and unnecessary or incorrect treatment. It is very important to distinguish it from other inflammatory diseases, especially FMF.^16^ Currently available criteria remain inadequate in making an accurate diagnosis of this common disease, and it is clear that new information is required to support the diagnosis. CRP and procalcitonin levels in PFAPA syndrome were examined by Yazgan et al.^14^ They found that patients with PFAPA diagnoses experienced fever episodes during which CRP values significantly increased but PCT values remained within normal ranges.^14^ In our study, ESR and CRP values were higher in the PFAPA group. In order to distinguish exudative tonsillitis caused by group A streptococcus and Epstein‐Barre virus from PFAPA attacks, Kanık et al.^17^ looked at the practical use of procalcitonin, ESR and CRP. They found that PFAPA and GAS could be identified more accurately using CRP and ESR than EBV tonsillitis.^17^ The results were similar to our study, but the number of cases was lower and the age of patients was different to that in our study. To further understand the pathophysiology of this pediatric illness, Brown et al.^18^ sought to profile blood cell and serum cytokine levels during the afebrile and febrile stages of periodic fever, aphthous stomatitis, pharyngitis, and adenitis (PFAPA) syndrome. They included a group of individuals with a median age of 4.9 years who were suffering “typical PFAPA” episodes.^18^ Although T cell‐associated cytokines IL7 and IL17 were repressed both during afebrile and febrile states, they found that IFN‐induced cytokine IP10/CXCL10 was elevated following the onset of fever.^18^ We did not look at these parameters, maybe they can be evaluated in future studies. The disease's cause is unknown; however, it is thought to be related to a process that is accompanied by an aberrant immune response and has been connected to an immune response failure. There was evidence of elevated IL‐1 levels during PFAPA episodes. In another trial, they administered a recombinant IL‐1 receptor antagonist to five PFAPA patients in light of the data that IL‐1 activation occurs during PFAPA flare‐ups. Their findings imply that when PFAPA flare‐ups, complement and IL‐1/‐18 are environmentally stimulated to become active, Th1 chemokines are produced, and activated T cells are then retained in peripheral tissues. With IP‐10/CXCL10 acting as a possible biomarker, IL‐1 inhibition may therefore be helpful for treating PFAPA attacks.^19^ Recurrent infection episodes may also be a factor in the progression of the illness.^20^ Inflammatory indicators can be useful in the diagnosis due to probable inflammatory processes that are inherent in the illness. The inflammatory markers NLR and MPV, which are collected as part of a complete blood count and utilized regularly in everyday practice, are affordable and simple to obtain. Increased neutrophil count or reduced lymphocyte count might cause elevated NLR. Acute or chronic inflammation is linked to circulating neutrophils.^21^, ^22^ NLR has been investigated in inflammatory cancers and auto‐inflammatory diseases where inflammatory processes also play a role, including familial Mediterranean fever, juvenile idiopathic arthritis, and systemic lupus erythematosus. It has been suggested that NLR may be an inflammatory marker in these conditions.^23^ The NLR rate was greater in instances of tonsillitis, and the sensitivity for tonsillitis was 0.62. It was observed that with a 3.24 cutoff point, the level of sensitivity and specificity were 62% and 59%, respectively. But, we did not compare with healthy children, maybe compared to healthy children, NLR rates in cases with PFAPA may be found to be higher. It is stated that MPV values in peripheral blood are seen to change in inflammatory processes. MPV therefore has been considered for use as an inflammatory marker, and evidence was obtained that MPV can be an indicator of auto‐inflammatory disorders.^23^ In a study from our country, the role of MPV in PFAPA syndrome was investigated and patients with PFAPA had lower MPV levels during an attack and during a time without an episode than those seen in controls.^23^ However, we did not find a significant relationship with MPV in our study. In another trial, 28 PFAPA syndrome patients who showed up at their clinic were investigated.^24^ Healthy kids who visited the general pediatrics polyclinic at the same age and sex as the sick group were enlisted as the control group.^24^ When patients with PFAPA syndrome were compared to the control group during the times between attacks, their mean platelet volumes (MPV) values were considerably lower than those of the control group.^24^ In our study, the MPV value was lower in the PFAPA group, but we could not find a statistical difference.

We also have some limitations. Our most important limitation is that our study is a single center and the number of cases is low. Another limitation was that the frequency of clinical findings for PFAPA was not noted. It would be useful to give what symptoms were expressed by patients (i.e., % with pharyngitis, % adenitis, % apthous stomatitis, etc). It would be better to propose a future studies to clarify how this predictive modeling of PFAPA diagnosis can be applied in other populations.

## 5 CONCLUSION

In conclusion, in cases where the age is >49 months, simple laboratory parameters can be used in the differential diagnosis of PFAPA syndrome and tonsillitis. The use of these parameters in particular can prevent the unnecessary use of antibiotics. CRP and ESR are the most important parameters in confirming a diagnosis and distinguishing PFAPA from acute tonsillitis. Again, we should keep in mind that the NLR rate will be higher in cases with tonsillitis. With simple laboratory parameters, PFAPA syndrome can be differentiated from a diagnosis of tonsillitis. This may reduce the costs associated with unnecessary antibiotic use. However, these findings still need to be confirmed by other future studies. Another practical question is will this difference in the labs between PFAPA and strep tonsillitis provide more than obtaining a strep culture? Perhaps not, but where opportunities are limited and access to strep cultures is limited, the results of this study can be instructive.

## AUTHOR CONTRIBUTIONS

Conceptualization, methodology, formal analysis and investigation, writing – review and editing: HO and AO.

## CONFLICT OF INTEREST STATEMENT

There is no conflict of interest in this study.

## Data Availability

All the data related to this work are available at the corresponding author.
